# Environmental Atlas of Prokaryotes Enables Powerful and Intuitive Habitat-Based Analysis of Community Structures

**DOI:** 10.1016/j.isci.2020.101624

**Published:** 2020-09-29

**Authors:** Kazumori Mise, Wataru Iwasaki

**Affiliations:** 1 Department of Biological Sciences, Graduate School of Science, The University of Tokyo, 2-11-16 Yayoi, Bunkyo-ku, Tokyo 113-0032, Japan; 2 Department of Computational Biology and Medical Sciences, Graduate School of Frontier Sciences, The University of Tokyo, 5-1-5 Kashiwanoha, Kashiwa, Chiba 277-8568, Japan; 3 Atmosphere and Ocean Research Institute, The University of Tokyo, 5-1-5 Kashiwanoha, Kashiwa, Chiba 277-8564, Japan; 4 Institute for Quantitative Biosciences, The University of Tokyo, 1-1-1 Yayoi, Bunkyo-ku, Tokyo 113-0032, Japan; 5 Collaborative Research Institute for Innovative Microbiology, The University of Tokyo, 1-1-1 Yayoi, Bunkyo-ku, Tokyo 113-0032, Japan

**Keywords:** Environmental Biotechnology, Microbiology, Microbial Genomics, Bioinformatics

## Abstract

The recent prevalence of high-throughput sequencing has been producing numerous prokaryotic community structure datasets. Although the trait-based approach is useful to interpret those datasets from ecological perspectives, available trait information is biased toward culturable prokaryotes, especially those of clinical and public health relevance, and thus may not represent the breadth of microbiota found across many of Earth's environments. To facilitate habitat-based analysis free of such bias, here we report a ready-to-use prokaryotic habitat database, ProkAtlas. ProkAtlas comprehensively links 16S rRNA gene sequences to prokaryotic habitats, using public shotgun metagenome datasets. We also developed a computational pipeline for habitat-based analysis of given prokaryotic community structures. After confirmation of the method effectiveness using 16S rRNA gene sequence datasets from individual genomes and the Earth Microbiome Project, we showed its validness and effectiveness in drawing ecological insights by applying it to six empirical prokaryotic community datasets from soil, aquatic, and human gut samples.

## Highlights

• We developed a database, ProkAtlas, denoting the habitat preferences of prokaryotes
• ProkAtlas represents a prokaryotic community using habitat preferences of its members
• The powerfulness of ProkAtlas is showcased by six datasets from various environments
• We provide web interface of ProkAtlas at https://msk33.github.io/prokatlas.html

## Graphical Abstract

**Figure:**
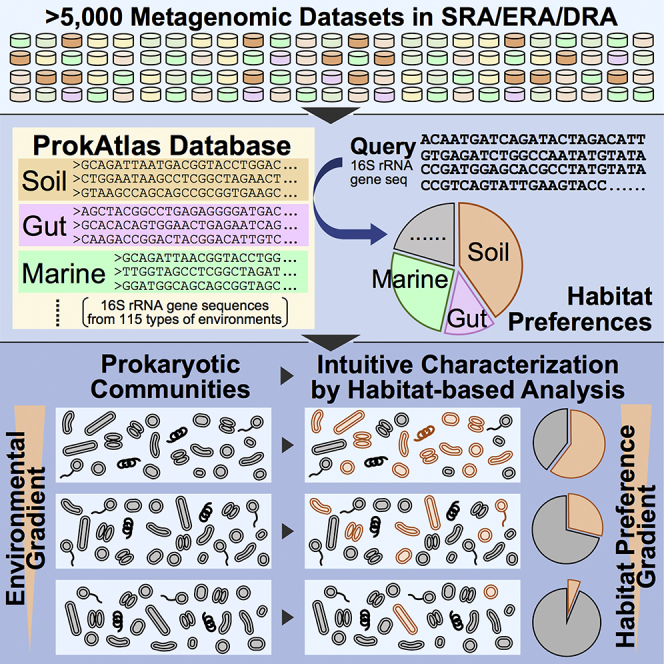

## Introduction

In the era of high-throughput sequencing, huge numbers of prokaryotic community structure datasets are being produced by 16S rRNA gene amplicon and shotgun metagenomic sequencing methods (Ramirez et al., 2018; Thompson et al., 2017). These abundant datasets from diverse environments have contributed to unveiling the diversity and distributions of environmental microbial communities on Earth (Delgado-Baquerizo et al., 2018; Gilbert et al., 2018; Sunagawa et al., 2015); however, such datasets still severely hamper microbial ecologists from intuitive interpretation. Each community structure dataset usually contains hundreds or thousands of operational taxonomic units, species, or genera (Lynch and Neufeld, 2015; Nemergut et al., 2013; Sogin et al., 2006), and it is also common to be presented as compositions of high-rank taxonomies (i.e., phyla or classes). Whether such dimensionality reduction is conducted or not, it is difficult to directly obtain interpretable ecological insights from community structure datasets, because ecological and physiological knowledge is not necessarily available for each of numerous members and such traits are often unconserved within high-rank clades (Martiny et al., 2015).

As a promising solution to this problem, the trait-based approach aims to enable the ecological interpretation of community structure datasets (Kearns and Shade, 2017; Krause et al., 2014). The basic idea of this approach is to first classify prokaryotes according to their ecological or physiological traits and then project that trait information to community structure datasets. To date, genome size (Barberán et al., 2014), rRNA gene copy number (Nemergut et al., 2016), growth rate, stress tolerance, capability to acquire carbon sources (Malik et al., 2020), metabolic potential (Louca et al., 2016), and pigmentation (Choudoir et al., 2018) data have been adopted for trait-based analyses of prokaryotic community structure datasets. Although these approaches performed well, their trait data were limited and biased to cultured prokaryotes with available genomic and physiological data. Prokaryotic communities, however, contain approximately 80% to more than 90% of uncultured members (Schloss et al., 2016; Steen et al., 2019).

As a promising trait-based approach that averts this issue, some studies exploited prokaryotic habitat information (Morton et al., 2017; Thompson et al., 2017; Washburne et al., 2017). Species that inhabit seawater are more likely to have the trait of adaptability to saline environments than species that inhabit freshwater only. Likewise, species that inhabit animal gut are more likely to have the trait of adaptability to copiotrophic (eutrophic) environments than species that inhabit soil only (here we use the term trait because of the affinity to the concept of the trait-based approach, although habitat preference itself may be defined as a characteristic rather than a functional or physiological trait). Importantly, habitat preferences of each prokaryote can be obtained by comparing metagenomic datasets from different environmental samples without the reliance on cultivation and isolation experiments.

In this study, we propose an intuitive and user-friendly method for habitat-based analysis and show its effectiveness in interpreting and investigating prokaryotic community structure datasets. We developed a database named ProkAtlas that links 16S rRNA gene sequences mined from metagenomes to prokaryotic habitats by substantially extending MetaMetaDB, which was previously developed by our group (Haider et al., 2018; Yang and Iwasaki, 2014). ProkAtlas now contains Illumina-sequencer datasets, considers differences in the sizes of research projects, and uses only shotgun metagenomic datasets to avoid biases due to the use of universal and/or clade-specific primers in amplicon sequencing (Klindworth et al., 2012) (note that ProkAtlas can be adopted for *analysis* of amplicon-sequencing datasets). We also developed the ProkAtlas pipeline for the habitat-based analysis of community structure datasets. As proofs of concept, we further drew a bird's-eye network of prokaryote co-occurrences among diverse environments and analyzed datasets from the Earth Microbiome Project (EMP), soil and lake-water samples with salinity gradients, human infant gut and glacial chronosequence soil samples undergoing primary successions, and river-water samples potentially polluted with pathogenic bacteria.

## Results and Discussion

### ProkAtlas Database and Pipeline

**Figure 1:**
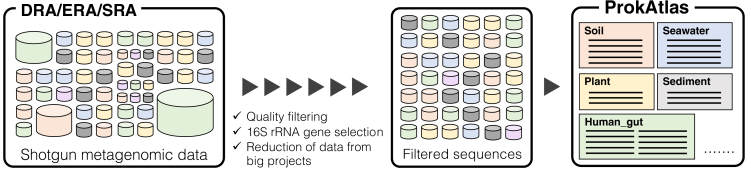
Schematic Illustration of ProkAtlas Construction The symbol colors and sizes represent the sample sources and data sizes, respectively.

ProkAtlas was developed as a comprehensive database of prokaryotic habitat traits based on a meta-analysis of metagenome shotgun sequencing datasets (Figure 1). Unlike EMP, ProkAtlas is free of PCR bias and applicable to any (variable) region of partial/full-length 16S rRNA gene sequences (note that biases from other experimental factors such as DNA extraction methods and library preparation processes would remain). It comprises 361,474 16S rRNA gene sequences from 5,368 shotgun metagenome projects registered in the INSDC SRA/ERA/DRA databases. Notably, to achieve reliable but efficient prokaryotic habitat estimation, we tried to balance the database comprehensiveness and size. As we show later, increasing the size of ProkAtlas marginally affects or improves the performance of habitat preference prediction, whereas computational cost linearly increases. It is also notable that the number of 16S rRNA gene sequences in ProkAtlas is comparable to those in Greengenes and SILVA (Glöckner et al., 2017; McDonald et al., 2012). Each sequence in ProkAtlas is labeled with one of the environmental categories listed in Table 1 for prokaryotic habitat estimation with 16S rRNA gene sequences (see Table S1 for the list of corresponding NCBI taxon IDs). Although NCBI taxon IDs contain environmental categories of different granularity, they are accompanied by all the metagenomic samples in DRA/ERA/SRA and therefore suitable for constructing a database that covers a wide variety of environments. The four major environmental categories, *soil*, *marine*, *freshwater*, and *rhizosphere*, comprise 55.3% of all sequences, and the top 26 categories comprise more than 90% of all sequences.

**Table 1:** Environmental Categories, Numbers of 16S rRNA Gene Sequences Labeled by These Categories, and Numbers of Research Projects of These Categories Contributing to ProkAtlas

Environmental Category	Number of Sequences	Number of Projects
Activated_carbon	44	1
Activated_sludge	7,383	78
Air	300	3
Algae	308	4
Anaerobic_digester	500	5
Annelid	1,038	13
Ant	300	3
Aquatic	2,667	29
Aquifer	1,900	19
Bat	143	2
Beach_sand	100	1
Biofilm	300	3
Biofilter	100	1
Biogas_fermenter	600	6
Bioreactor	2,923	32
Bioreactor_sludge	200	2
Biosolids	200	2
Bird	100	1
Bovine	200	2
Bovine_gut	700	7
Cave	100	1
Chicken_gut	731	9
Compost	500	5
Coral	1,100	11
Crab	100	1
Crustacean	100	1
Endophyte	30	1
Epibiont	100	1
Estuary	200	2
Feces	1,306	14
Fermentation	300	3
Fish_gut	25	1
Food	604	7
Food_fermentation	300	3
Food_production	100	1
Fossil	200	2
Freshwater	43,216	437
freshwater_sediment	13,744	239
Fungus	3,737	38
Glacier	500	5
Groundwater	9,540	97
Gut	3,167	36
Halite	14	1
Hot_springs	1,056	12
Human_bile	111	2
Human_blood	1	1
Human_eye	131	2
Human	1,815	25
Human_gut	7,502	80
Human_lung	291	3
Human_oral	500	5
Human_reproductive_system	100	1
Human_skin	400	4
Hydrocarbon	33	1
Hydrothermal_vent	4,016	44
Hypersaline_lake	900	9
Hypolithon	113	2
Indoor	100	1
Insect	241	4
Insect_gut	200	2
Invertebrate	300	3
Lake_water	5,700	57
Landfill	400	4
Leaf	500	5
Lichen	500	5
Marine	45,298	461
Marine_sediment	3,281	35
Microbial_fuel_cell	100	1
Microbial_mat	1,561	17
Mine_drainage	200	2
Mine_tailings	200	2
Mixed_culture	100	1
Money	200	2
Mosquito	180	2
Moss	600	6
Mouse_gut	1,194	18
Oil_field	3	1
Oral	66	1
Oyster	100	1
Paper_pulp	100	1
Parasite	26	1
Peat	7,683	77
Permafrost	1,449	16
Phyllosphere	12,100	121
Pig_gut	576	6
Plant	4,579	53
Plastic	6	3
Pollen	200	2
Rat_gut	100	1
Rhizosphere	21,152	213
Rice_paddy	3,100	31
Rock	148	2
Rock_porewater	100	1
Root_associated_fungus	100	4
Root	400	1
Salt_lake	1,900	19
Salt_marsh	5,400	54
Sea_squirt	400	4
Seawater	2,977	30
Sediment	6,431	66
Skin	100	1
Sludge	100	1
Soil	90,158	984
Sponge	300	3
Stromatolite	100	1
Subsurface	3,020	32
Surface	100	1
Symbiont	69	1
Termite_gut	2,919	31
Terrestrial	2,134	22
Tick	100	1
Urban	6	1
Viral	600	6
Wastewater	3,228	35
Wetland	11,900	119

The environmental categories were based on annotations in the NCBI SRA database. Note that due to data processing, several environmental categories are associated with a few sequences. See also Tables S1, S2, and S3; Figure S7.

We further developed a pipeline to estimate the habitats of prokaryotes based on 16S rRNA gene sequences using ProkAtlas. Basically, this pipeline uses a sequence similarity search to query every 16S rRNA gene sequence of given data against the ProkAtlas database and obtains a list of environmental categories that are labeled to the hit sequences. Compositions of the retrieved environmental categories are represented by habitat preference scores. Note that (nearly) identical sequences are included within the 361,474 sequences in ProkAtlas and one query sequence can be mapped to two or more environmental categories. Because of this one-to-many relationship, the habitat preference scores are presented as compositions of multiple environmental categories. More details on the pipeline are available in Transparent Methods and Figures S1 and S2.

### Bird's-Eye Visualization of Prokaryote Co-occurrence Network among Diverse Environments

**Figure 2:**
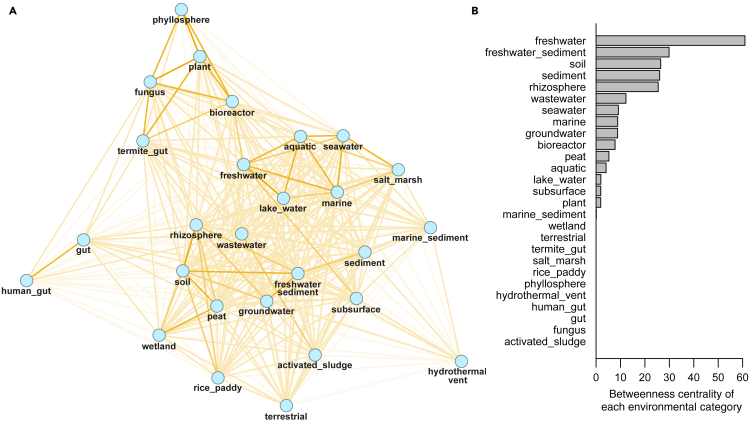
Network Analysis of Co-occurrences of Prokaryotes among Environments (A) A bird's-eye network visualization of the co-occurrences. Nodes represent environmental categories. Edges are drawn only if Bray-Curtis dissimilarities are less than 0.9 (smaller dissimilarities shown by darker colors). (B) A bar chart showing the betweenness centrality of each environment (i.e., the number of node pairs whose shortest paths contain the node representing that environment).

All ProkAtlas sequences were mapped to 60,278 SILVA entries, and their composition in each environmental category was quantified. By enumerating 16S rRNA gene sequences that co-occur in different environments using ProkAtlas, we obtained a comprehensive view of co-occurrences of prokaryotes among diverse environments as a network (Figure 2A).

As expected, related environments such as *soil* and *rhizosphere* were strongly associated with each other. The betweenness centrality, which quantifies the propagation of prokaryotes among different environmental categories, of extreme environment (i.e., *hydrothermal_vent*) was low (Figure 2B). Regarding this observation, it is reasonable that prokaryote co-occurrences or migrations via extreme environments are rare because of their non-moderate conditions and geographical isolation. It was also found that betweenness centralities of host-associated environments were relatively low. This observation was rather unexpected because prokaryotic hosts, especially animals, may bring prokaryotes to different environments and promote their migration (Grossart et al., 2010). We assume that strong prokaryote-host dependencies prohibit prokaryotes from settling in new environments, regardless of their hosts' movement, and that prokaryotic hosts may actually have limited roles in shaping microbial distributions across the earth.

### Consistency between Sources of Isolated and Non-isolated Prokaryotes and ProkAtlas Habitat Estimation

**Figure 3:**
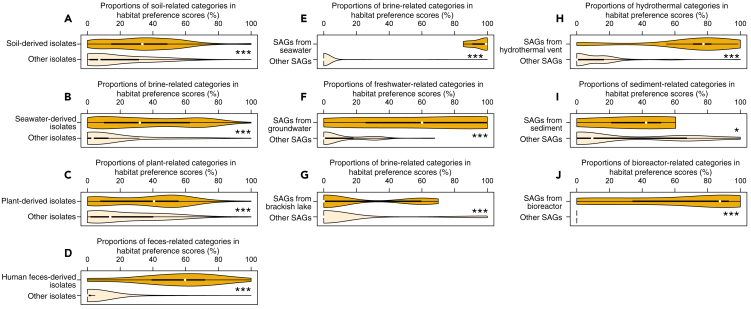
Habitat Preference Scores of Isolates/SAGs Derived from a Specific Type of Environment Each of the darker orange plots indicates the scores of isolates/SAGs from the relevant environment, whereas the lighter orange one indicates those of the other isolates/SAGs. A thick black line and a white circle indicate the 25–75% range and median within each plot, respectively. (A) Prokaryotic isolates' habitat preference scores of soil-related environments (*soil*, *rhizosphere*, *rice_paddy*, and *wetland*). (B) Prokaryotic isolates' habitat preference scores of brine-related environments (*marine*, *salt_marsh*, and *seawater*). (C) Prokaryotic isolates' habitat preference scores of plant-associated environments (*rhizosphere*, *phyllosphere*, *root*, *soil*, and *plant*). (D) Prokaryotic isolates' habitat preference scores of feces-associated environments (*feces*, *gut*, and *human_gut*). (E) SAGs' habitat preference scores of brine-related environments (*marine*, *salt_marsh*, *salt_lake*, and *seawater*). (F) SAGs' habitat preference scores of freshwater-related environments (*freshwater*, *lake_water*, and *groundwater*). (G) SAGs' habitat preference scores of brackish lake-related environments (*marine*, *salt_marsh*, *salt_lake*, and *seawater*). (H) SAGs' habitat preference scores of hydrothermus-related environments (*hydrothermal_vent* and *hot_springs*). (I) SAGs' habitat preference scores of marine sediment-related environments (*freshwater_sediment*, *sediment*, *marine_sediment*, *marine*, *salt_marsh*, and *salt_lake*). (J) SAGs' habitat preference scores of bioreactor-related environments (*bioreactor* and *activated_sludge*). Asterisks denote the results of the Mann-Whitney U tests (∗p < 0.05, ∗∗∗p < 0.001) between each pair of violin plots. See also Figures S3 and S4.

ProkAtlas was applied to 1,021 (nearly) full-length 16S rRNA gene sequences of pure-isolated bacterial strains from the International Journal of Systematic and Evolutionary Microbiology (IJSEM) phenotypic database (Barberán et al., 2017), as well as to 201 16S rRNA gene sequences retrieved from a large single amplified genome (SAG) sequencing project (Rinke et al., 2013). All sequences of pure isolates and 183 (91.0%) sequences from SAGs had one or more significant hits in ProkAtlas. We then calculated the habitat preference scores (see Methods) and found that these thes scores were overall consistent with their environmental sources: scores of soil-related environmental categories (namely *soil*, *rhizosphere*, *rice_paddy*, and *wetland*), for example, were significantly higher in soil-derived sequences compared with sequences of isolates from all the other environments (Figure 3A, Mann-Whitney U test, p < 0.001). This trend was similarly observed for various sets of environmental categories, including isolates and/or SAGs from seawater, plants, feces, groundwater, lake water, hydrothermal vent, and bioreactors (Figures 3B–3J). On the other hand, habitats estimated by ProkAtlas were inconsistent with the actual environmental sources for a portion of individual query sequences. Such conflict may be attributed to the fact that many prokaryotic species are distributed in broad ranges of environments (Sriswasdi et al., 2017) and isolation sources of cultured strains or sampling sites of SAGs could be actually rare habitats of that prokaryotic group. That means, although estimated habitat of a specific individual prokaryote can be sometimes incorrect, habitat preference scores of a prokaryotic community consisting of multiple species can still be an informative proxy of that community. In addition, the abovementioned trends were reproduced when the alignment length thresholds were raised to 200 or 250 bases (Figure S3). Because of this, we assume that 150-bp threshold (the default value in our pipeline) would be long enough to achieve overall accuracy, while retaining enough amount of significant hits.

### Habitat-Based Analysis of the EMP Dataset

To test the versatility of habitat-based prokaryotic community analysis using ProkAtlas, we reanalyzed the EMP dataset, a large community-based project that collects and analyzes prokaryotic community samples from various natural environments (Thompson et al., 2017). Because each of the 16S rRNA gene amplicon-sequencing datasets in the EMP dataset is tagged with sampling-site metadata described by EMP Ontology, this dataset was used for assessing the validity of ProkAtlas-based analysis.

**Figure 4:**
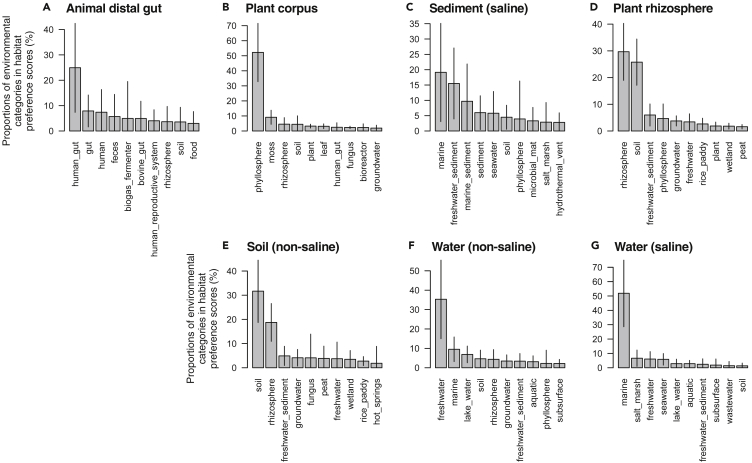
Habitat Preference Scores of EMP Prokaryotic Communities Habitat preference scores in each sampling site represented by EMP Ontology level 3 terms are shown. (A–G) (A) Animal distal gut, (B) plant corpus, (C) sediment (saline), (D) plant rhizosphere, (E) soil (non-saline), (F) water (non-saline), and (G) water (saline). The y axes show the proportions of environmental categories within individual estimated habitat compositions. Means and standard deviations (by error bars) among EMP samples are shown. See also Figure S5.

Among the 91,364 sub-operational taxonomic units (sOTUs) in the EMP dataset, 32,117 (35.2%), accounting for 65.3% of the total reads, were successfully mapped to ProkAtlas. The habitat preference scores of prokaryotic communities estimated by ProkAtlas were generally consistent with the sampling-site metadata in the EMP dataset (Figure 4). For example, prokaryotic communities annotated by *soil (non-saline)* showed higher habitat preference scores of *soil* (31.7% ± 12.9%, mean ± sd) and *rhizosphere* (18.7% ± 7.82%), compared with the other communities. Similarly, communities annotated as *water (saline)* showed higher habitat preference scores of *marine* (51.9% ± 23.3%). This result, in line with the habitat preference scores of sequences from isolates and SAGs, highlights the reliability of the habitat preference scores. This in turn means that environmental source of a given sample may be estimated by ProkAtlas, for example, to address forensic concerns (Carter et al., 2020).

When one of the five alternative datasets (sets A–E) was employed instead of ProkAtlas, the consequent habitat preference scores were only marginally affected (Figures S4 and S5), where larger-size databases (sets C–E) slightly improved the sequence coverages (51.5%–51.7% of sOTUs, accounting for 75.4%–76.8% of total reads, were mapped). We thus argue that the size of ProkAtlas achieves a good balance between information content and computational usability.

### Habitat-Based Analyses of Soil and Lake-Water Samples with Salinity Gradients

As a proof-of-concept of the habitat-based analysis of prokaryotic community structures, we applied ProkAtlas to six 16S rRNA gene amplicon-sequencing datasets (Table 2). The first dataset contained 124 agricultural soil samples with different salinities sampled at 31 sites in northwest China (Zhao et al., 2020). These sampling sites span more than 400 km in longitude, and four samples were obtained from each site. The dataset contained 12,094 sOTUs, among which 12,052 (99.6%) and 7,631 (63.1%), accounting for 99.8% and 67.9% of the total reads per sample on average, were taxonomically assigned at the phylum level and successfully mapped to ProkAtlas, respectively.

**Table 2:** Six 16S rRNA Gene Amplicon-Sequencing Datasets Reanalyzed Using Habitat-Based Approach

Sample Description	Number of Samples	Data Availability	Reference
Saline agricultural soils sampled at 31 points scattered over 400 km (north-west China)	124	INSD SRP136143	Zhao et al. (2020)
Saline and non-saline water samples sampled at 25 lakes (Tibet Plateau, China)	78	INSD PRJNA503775	Ji et al. (2019)
Stool of newborn Finnish infants (0–36 months old)	776	DIABIMMUNE project website	Yassour et al. (2016)
Bulk soil samples at different developmental stages, obtained along retreating glacier (Midtre Lovénbreen Glacier, Norway)	21	INSD PRJEB12640	Mapelli et al. (2018)
Bulk soil samples at different developmental stages, obtained along retreating glacier (Hailugou Glacier chronosequences, China)	21	INSD PRJNA354498	Jiang et al. (2018)
Water sampled along Manoa Stream (Hawaii, USA)	25	INSD PRJNA376213	Kirs et al. (2017)

**Figure 5:**
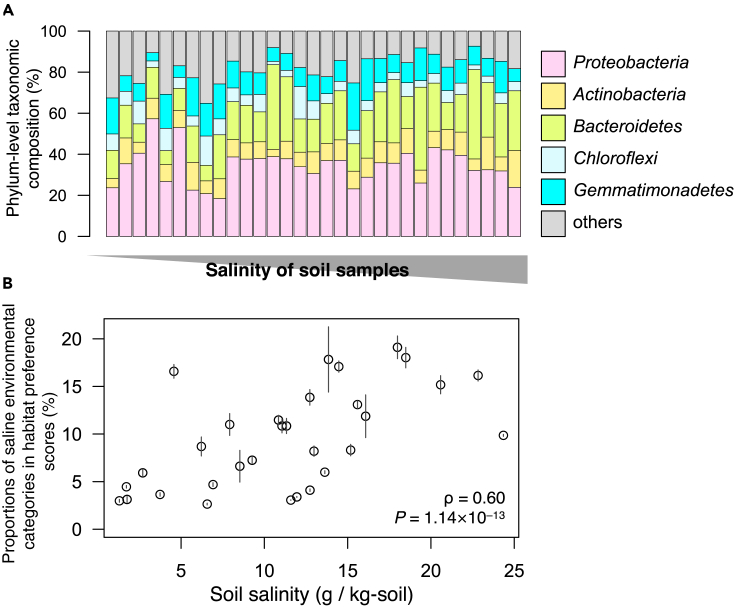
Habitat-Based Analysis of Soil Samples with Salinity Gradients (A) Phylum-level taxonomic structures of the 31 plots ordered by soil salinity concentration (higher on the right than on the left). Each bar denotes the average of four replicates within one plot. Means among four replicates for each plot are indicated. (B) A scattergram of soil salinity concentrations and sum of habitat preference scores of brine-related environmental categories (*estuary*, *hypersaline_lake*, *marine*, *salt_lake*, *salt_marsh*, *seawater*, and *marine_sediment*). Means and standard deviations (by error bars) among four replicates for each plot are indicated. Result of the Spearman's correlation test is also shown. See also Figure S6.

When phylum-level taxonomic structures were investigated as many amplicon-sequencing studies do, the estimated compositions were dominated by the phyla Proteobacteria (34.3% ± 8.99%, mean ± SD), Bacteroidetes (20.8% ± 9.47%), and Gemmatimonadetes (11.7% ± 4.63%) (Figure 5A). On the other hand, when the estimated habitats were investigated, we observed a clear trend that the habitat preference compositions were affected by soil salinity concentration (Figure 5B). More specifically, saline environments such as *marine, seawater*, *estuary*, and *salt_lake* showed substantial variation among the samples (2.44%–26.4%) and a significant positive correlation with the soil salinity concentration (Spearman's correlation test, ρ = 0.60, p < 0.001) as expected. Thus, ProkAtlas clearly and intuitively highlights the microbial community characteristics of high-salinity soils, which is consistent with previous knowledge on the relationship between salinity and prokaryotic community structures (Lozupone and Knight, 2007; Rath et al., 2019).

**Figure 6:**
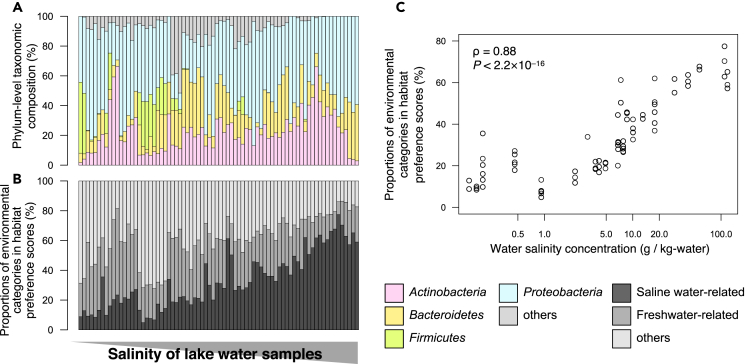
Habitat-Based Analysis of Saline and Non-saline Water Samples from 25 Lakes (A) Phylum-level taxonomic structures of the 76 samples ordered by salinity (higher on the right than on the left). (B) Habitat preference scores. Saline water-related: *hypersaline_lake*, *marine*, *marine_sediment*, *salt_lake*, *salt_marsh*, and *seawater*. Freshwater-related: *aquifer*, *freshwater*, *freshwater_sediment*, *groundwater*, and *lake_water*. (C) A scattergram of water salinity concentrations and sum of habitat preference scores of saline water-related environmental categories in the estimated habitat compositions (*estuary*, *hypersaline_lake*, *marine*, *marine_sediment*, *salt_lake*, *salt_marsh*, and *seawater*). Result of the Spearman's correlation test is shown. See also Figure S6.

The second dataset contained saline and non-saline lake water samples (Ji et al., 2019), totaling 78 samples from 25 lakes with diverse salinity from the Tibetan Plateau. Two of the samples lacking sampling site information were excluded from analysis. The dataset contained 6,054 sOTUs, among which 5,241 (86.6%), accounting for 91.2% of the total reads per sample, on average were successfully mapped to ProkAtlas. All the sOTUs were taxonomically assigned at the phylum level. All samples were dominated by phyla Actinobacteria, Bacteroidetes, Cyanobacteria, and/or Proteobacteria (Figure 6A). Phylum-level taxonomic compositions diverged highly even between samples with similar salinity concentrations and gave few ecological insights. On the other hand, the habitat-based analysis was able to differentiate the prokaryotic communities between the saline and non-saline lakes (Figure 6B). The proportions of saline-water-related categories were significantly and strongly correlated with salinity (Figure 6C, Spearman's correlation test, ρ = 0.88, p < 0.001). Here, ProkAtlas reproduces the effect of salinity on prokaryotic community structures, which was previously elucidated at global and local scales (Lozupone and Knight, 2007; Rath et al., 2019; Thompson et al., 2017), highlighting the robustness and validity of the database and pipeline. In addition, ProkAtlas relabels prokaryotic community members as salinity-tolerant/sensitive and thereby facilitates intuitive interpretation of prokaryotic community structures without cumbersome calibration or modeling.

### Habitat-Based Analyses of Infant Gut and Glacial Chronosequence Soil Samples

Next, the habitat-based analysis was applied to three datasets of prokaryotic communities under primary succession. The third dataset consisted of human infant gut microbiome samples (Yassour et al., 2016). In this study, 654 time-series infant feces samples were collected from Finnish infants aged 2 to 36 months. The dataset contained 2,113 sOTUs, among which 2,113 (100%) and 1,374 (65.0%), accounting for 100% and 96.8% of total reads per sample on average, respectively, were taxonomically assigned at the phylum level and successfully mapped to ProkAtlas.

**Figure 7:**
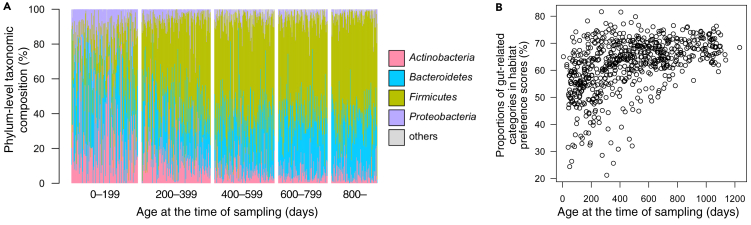
Habitat-Based Analysis of Human Infant Gut Microbiome Samples (A) Phylum-level taxonomic structures of the 654 samples ordered by sampling ages (older on the right). (B) A scattergram of infant ages and sum of habitat preference scores of human gut-related environmental categories (*gut* and *human_gut*). See also Figure S6.

When phylum-level taxonomic compositions were investigated, the compositions were highly diverse until approximately 400 days after birth, after which the compositions stabilized and were dominated by Firmicutes and Bacteroidetes (Figure 7A). Although this process was already well known (Bäckhed et al., 2015), the habitat-based analysis gave another view on the process as the convergence to *human gut-related* environmental categories (Figure 7B). This pattern is consistent with the process that infant gut microbiome undergoes temporal successions toward a stable and “matured” state. It may also be notable that recent trait-based studies in microbial ecology have suggested that, whereas taxonomic compositions during primary and secondary successions are often stochastic and uninterpretable (Ferrenberg et al., 2013), traits of prokaryotic communities follow a path that is more predictable and interpretable (Kearns and Shade, 2017; Nemergut et al., 2016).

**Figure 8:**
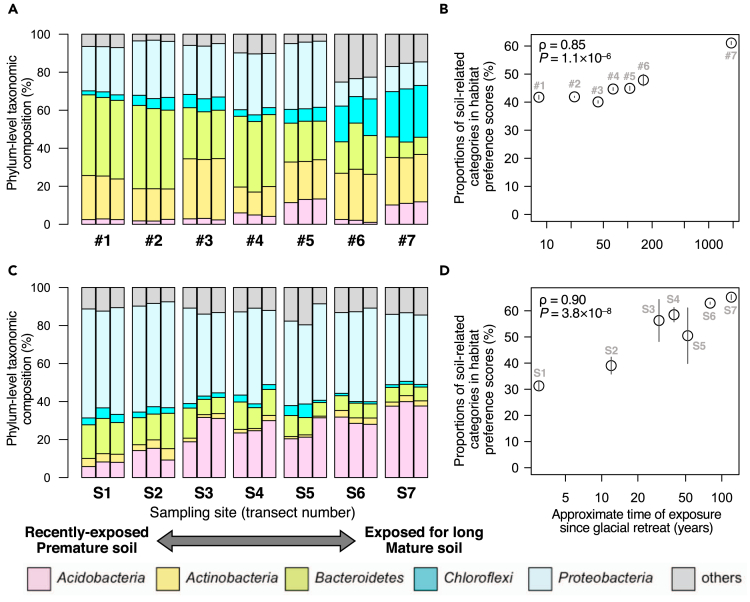
Habitat-Based Analysis of Glacial Chronosequence Soil Samples (A and B) (A) Phylum-level taxonomic structures and (B) sum of habitat preference scores of estimated soil-related environmental categories (*soil*, *rhizosphere*, *rice_paddy*, and *wetland*) in soil samples taken from Midtre Lovénbreen Glacier moraine (Norway) (Mapelli et al., 2018). (C and D) (C) Phylum-level taxonomic structures and (D) sum of habitat preference scores of estimated soil-related environmental categories (*soil*, *rhizosphere*, *rice_paddy*, and *wetland*) in soil samples taken from Hailuogou Glacier chronosequence (China) (Jiang et al., 2018). The samples are ordered by the length of weathering time. In (B) and (D), means and standard deviations (by error bars) among three replicates for each plot are shown, along with results of the Spearman's correlation tests. See also Figure S6.

The fourth and fifth datasets contained glacial chronosequence soil samples from two different glaciers (Jiang et al., 2018; Mapelli et al., 2018), which provide transects at different developmental stages from unweathered bedrocks to matured (extensively weathered) soil (Castle et al., 2017; Delgado-Baquerizo et al., 2019). Each of these datasets contained 21 bulk soil samples collected at seven sampling sites along a retreating glacial chronosequence. One was from Midtre Lovénbreen Glacier moraine (Norway) (Mapelli et al., 2018), and the other was from Hailuogou Glacier chronosequence (China) (Jiang et al., 2018). After the glacial retreat, those sites have been exposed to soil weathering for different time lengths. In both datasets, the phylum-level taxonomic compositions along the chronosequences presented clear gradients; however, the taxonomic clades constituting the gradients were quite different between the two—they were phyla Bacteroidetes and Chroloflexi in the Norwegian dataset (Figure 8A) but phyla Acidobacteria, Bacteroidetes, and Proteobacteria in the Chinese dataset (Figure 8C). Both of the gradients could be the outcomes of chemical condition changes (e.g., phosphorus depletion mitigation as a result of weathering) (Castle et al., 2017; Delgado-Baquerizo et al., 2017); however, their apparently different patterns hamper unified understanding of the prokaryotic community successions. On the other hand, the habitat-based analysis of the prokaryotic communities clearly illustrated similar convergence to soil-related environments during the courses of pedogenesis in both sites (Figures 8B and 8D). This suggests that prokaryotic habitat preference can be a useful trait for analyzing community successions. In addition, a notable difference was seen between the results of the bulk soil and infant gut datasets. Many of the infant gut prokaryotic communities were “matured” from the beginning possibly due to the priority effect (Figure 7B) in contrast to the soil prokaryotic communities (Figures 8B and 8D).

In summary, the three datasets indicate that ProkAtlas can be used to evaluate the maturity of prokaryotic ecosystems undergoing temporal successions, without prior investigation on what the “matured” state is like. Notably, such effectiveness of habitat-based analysis can be placed into the context of recent discussions on trait-based microbial community ecology: although the primary or secondary successions of microbial communities are often stochastic and unpredictable (Ferrenberg et al., 2013), trait-based patterns tend to be more conserved, predictable, and easier to interpret (Kearns and Shade, 2017; Nemergut et al., 2016).

### Habitat-Based Analyses of Potentially Polluted River Water

Finally, the habitat-based analysis was applied to prokaryotic community mixture from distinct environments. The sixth dataset contained potentially polluted river-water samples (Kirs et al., 2017). In this study, 25 water samples were collected at nine sampling sites in the Manoa Stream, which flows through urbanized areas on Oahu Island, Hawaii, USA. High levels of fecal indicator bacteria (FIB) were reported in the estuary of Manoa stream neighboring popular bathing beaches (Goto and Yan, 2011), and sources of FIB were of interest in the contexts of both environmental and health sciences. The dataset contained 4,061 sOTUs, among which 4,000 (98.5%) and 2,389 (58.8%), accounting for 99.7% and 75.1% of the total reads, were taxonomically assigned at the phylum and proteobacterial class levels and successfully mapped to ProkAtlas, respectively.

**Figure 9:**
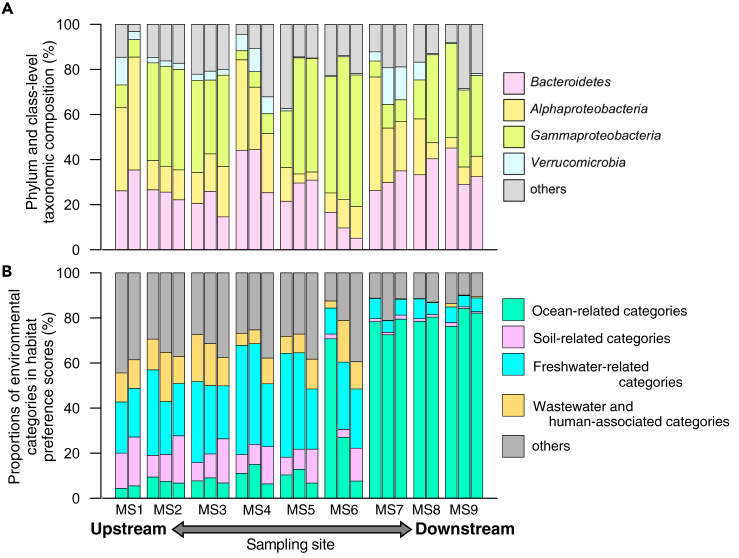
Habitat-Based Analysis of Potentially Polluted River-Water Samples (A) Phylum- and proteobacterial-class level taxonomic structures at nine sampling points (more downstream on the right than on the left). (B) Habitat preference compositions. Ocean-related categories: *estuary*, *hypersaline_lake*, *marine*, *salt_lake*, *salt_marsh*, *seawater*, and *marine_sediment*. Soil-related categories: *rhizosphere* and *soil*. Freshwater-related categories: *freshwater*, *aquifer*, *groundwater*, and *lake_water*. Wastewater and human-associated categories: *gut*, *human*, *human_gut*, and *wastewater*. The community structures are ordered by the geographical locations of sampling sites. See also Figure S6.

The taxonomic structures showed a clear gradient from upstream (MS1–5) to downstream samples (MS7–9) (Figure 9A). On the other hand, the investigation of the estimated habitats visualized two important ecological features. First, the transition from soil- and freshwater-related environments to seawater-related environmental categories was clearly observed from the upstream (MS1–5) to midstream (MS6) and downstream sites (MS7–9) (Figure 9B). MS7–9 are located in a canal that is connected to the sea and directly influenced by tides, whereas MS6 is located approximately 500 m upstream to the confluence with the canal (Kirs et al., 2017). Second, environmental categories related to anthropogenic water contamination (e.g., *human_gut* and *wastewater*) showed a decrease from the upstream to midstream sites. This result was rather unexpected because potential pollution was expected to be introduced in urbanized areas (MS2–5) and increase their compositions along the river flow. Instead, the habitat-based analysis suggests that river water in the upstream, conserved forest area (MS1) already contains FIB. Notably, in line with this interpretation, some studies have claimed that riverine FIB largely come from soil instead of human pollution (Goto and Yan, 2011; Kirs et al., 2017). We note that the source tracking methods (Knights et al., 2011; Unno et al., 2018) can also be adopted for a similar purpose, but ProkAtlas differs in that it is a ready-to-use and versatile database and users do not need to prepare reference datasets for themselves.

In summary, the habitat-based analysis here answered questions like “Where are prokaryotic communities from distinct environments mixed?” and “How do mixed communities develop in different environments?” without cumbersome preparation of reference datasets.

### Future Perspectives

In this study, we developed ProkAtlas for the habitat-based analysis of prokaryotic community structure datasets. The application of ProkAtlas to six datasets succeeded in intuitively highlighting environment-prokaryotic community structure relationships, delineating temporal succession patterns of prokaryotic communities and providing insights into environmental and ecological surveillance such as pollution sources. Notably, ProkAtlas is independent of biases toward cultured prokaryotes, which is a common problem in many tools in microbiology. In addition, as a comprehensive database, ProkAtlas is readily applicable to many prokaryotic community structure datasets without the need for additional experiments or cumbersome data processing and might also be applicable for discovering the general principles behind microbial evolution and migration. By complementing existing approaches, this habitat-based analysis will contribute to gaining more insights into various research areas, including microbial ecology, environmental science, evolutionary science, agriculture, health science, and medicine, by taking advantage of the ever-increasing prokaryotic community structure datasets.

### Limitations of the Study

A potential flaw of ProkAtlas is that it depends on environmental classifications defined by INSDC DRA/ERA/SRA. Although the use of this classification system enabled us to cover all the numerous metagenomic samples and enhanced the comprehensiveness of ProkAtlas, it is neither strictly defined nor hierarchically organized, and contains categories of different granularity. For example, metagenomic sequences from human gut samples may be labeled either as *human_gut* or *human*, and this choice is ultimately up to the data submitter. Similarly, sequences from rice field soils may be labeled either as *soil* or *rice_paddy*. Nevertheless, we did not adopt previously developed environmental ontologies such as MIGS/MIMS and EnvO in the present study because INSDC contains many samples lacking these systematic annotations. Here, for the sake of readers, we present an example of classification of environmental categories by referring to EMP Ontology and a supervised machine-learning of abstract texts attached to each project in DRA/ERA/SRA (Figure S7). Although such systematic annotations would restructure ProkAtlas in the future, currently it is left to ProkAtlas users to select and merge categories depending on applications and interest.

### Resource Availability

#### Lead Contact

Further information and requests for resources should be directed to and will be fulfilled by the Lead Contact, Wataru Iwasaki (iwasaki@bs.s.u-tokyo.ac.jp).

#### Materials Availability

This study did not generate new unique reagents.

#### Data and Code Availability

The ProkAtlas database, pipeline, and the source code for calculating habitat preference scores are available at https://msk33.github.io/prokatlas.html.

## Methods

All methods can be found in the accompanying Transparent Methods supplemental file.

## Supplemental Information

Document S1. Transparent Methods and Figures S1–S7

Table S1. Names and NCBI Taxon IDs of Environmental Categories Associated with Metagenomic Data in the GenBank SRA Database, Related to Table 1 and the Transparent Methods

Table S2. Accession IDs and Sequence Attributes of the 5,368 BioProjects Constituting ProkAtlas and the 48,056 Runs under these BioProjects, Related to Table 1 and the Transparent Methods

Table S3. Accession IDs and Sequence Attributes of the 11,148 Runs Constituting ProkAtlas, Related to Table 1 and the Transparent Methods
