# Supplementary material for: Environmental Atlas of Prokaryotes Enables Powerful and Intuitive Habitat-Based Analysis of Community Structures
Source: iScience. 2020 Sep 29;23(10):101624. doi: 10.1016/j.isci.2020.101624 (PMC7581931; doi:10.1016/j.isci.2020.101624)
Supplement: Document S1. Transparent Methods and Figures S1–S7 [file mmc1.pdf]

iScience, Volume *23*

Supplemental Information

Environmental Atlas of Prokaryotes Enables Powerful and Intuitive Habitat-Based Analysis of Community Structures

Kazumori Mise and Wataru Iwasaki

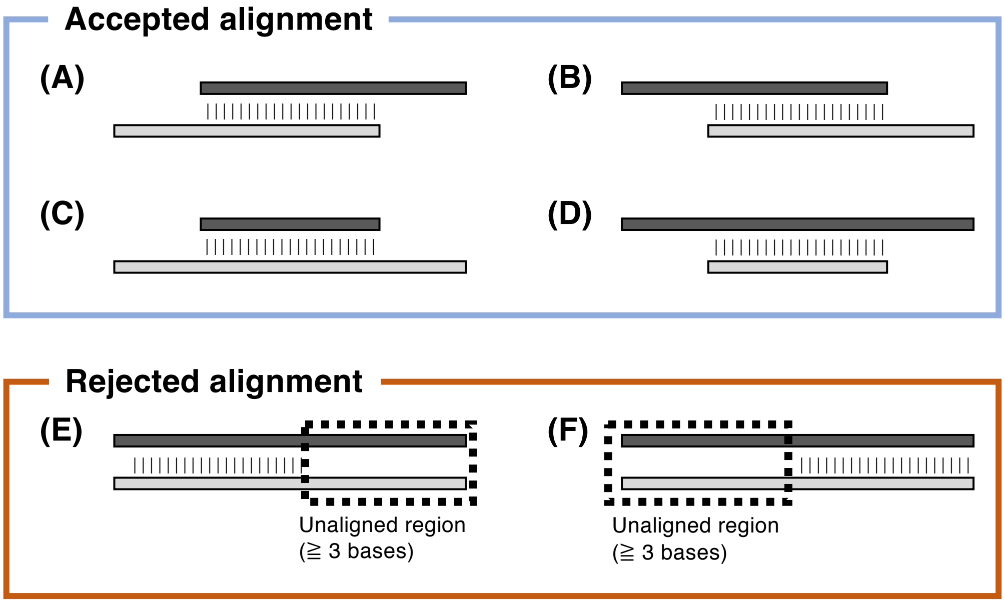

Figure S1. Schematic representation of alignment criteria of 16S rRNA gene sequences in the ProkAtlas pipeline, Related to the Transparent Methods

Black and gray rectangles indicate query and subject sequences, respectively. Vertical bars indicate successfully aligned regions by pairwise local alignment. While the upper four partial alignment patterns are accepted, the bottom two patterns are rejected.

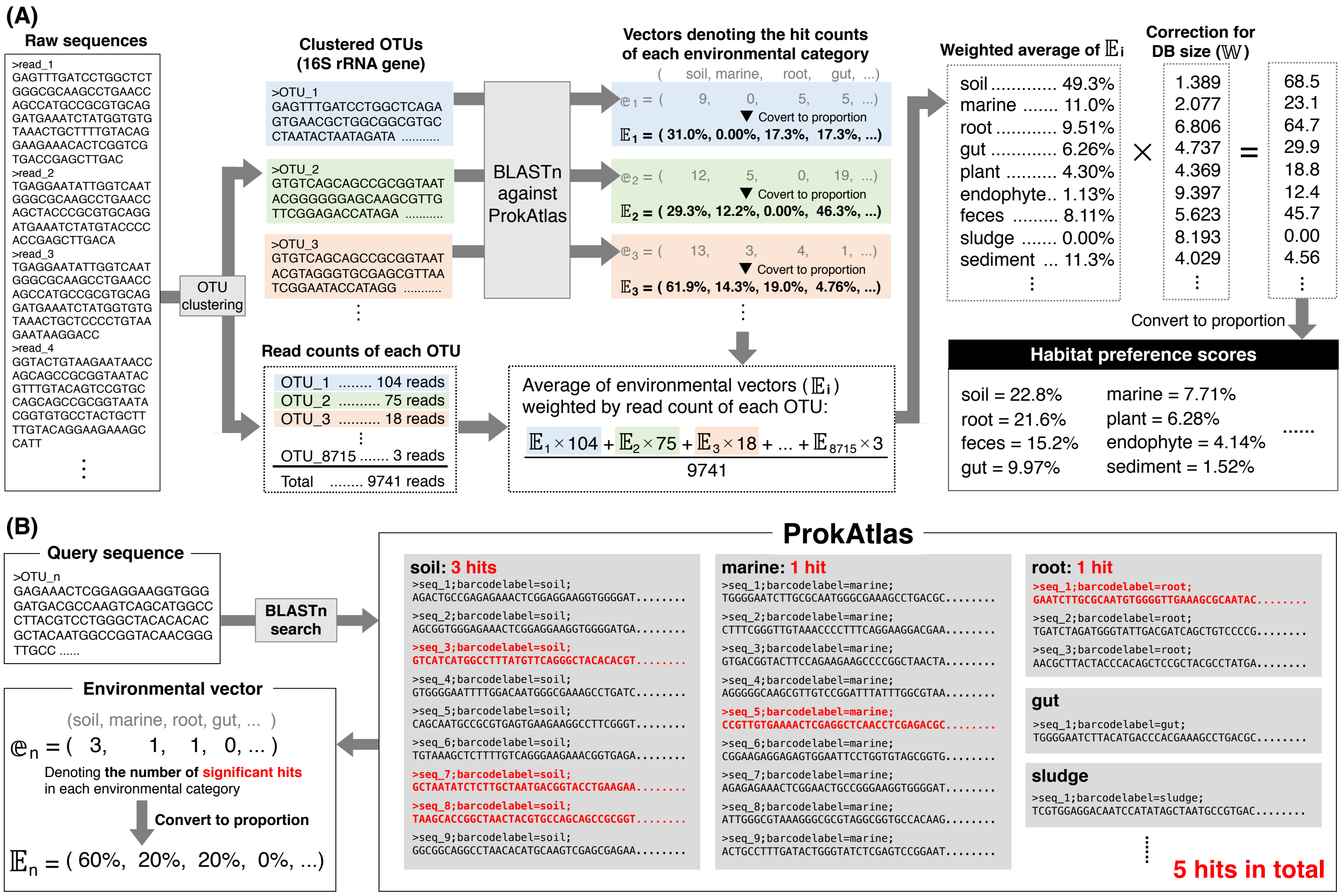

Figure S2. A schematic illustration of procedures to calculate habitat preference scores for prokaryotic communities, Related to the Transparent Methods

(A) Overview of the process. Prokaryotic community composition can be defined by the representative sequence and read count of each OTU. First, to characterize habitat preference of each OTU, the OTU representative sequences are subjected to BLASTn search against ProkAtlas database. Typically, each OTU has significant hits to sequences in multiple environmental categories, and the habitat preferences of each OTU may be represented as the environmental vector $\mathbb{E}$. Overall habitat preference of a community is denoted as the average of $\mathbb{E}$ weighted by the read count of each OTU, followed by the correction for the overrepresentation of well-studied environments like *soil* and *marine*. (B) Detailed schema of "one-to-many" mapping of a query sequence on ProkAtlas database. If one query sequence has multiple hits in ProkAtlas, the number of hits for each environmental category is presented as $\mathbb{e}$. Therein, $\mathbb{e}$ is converted to the environmental vector $\mathbb{e}$ denoting the habitat preference of the OTU.

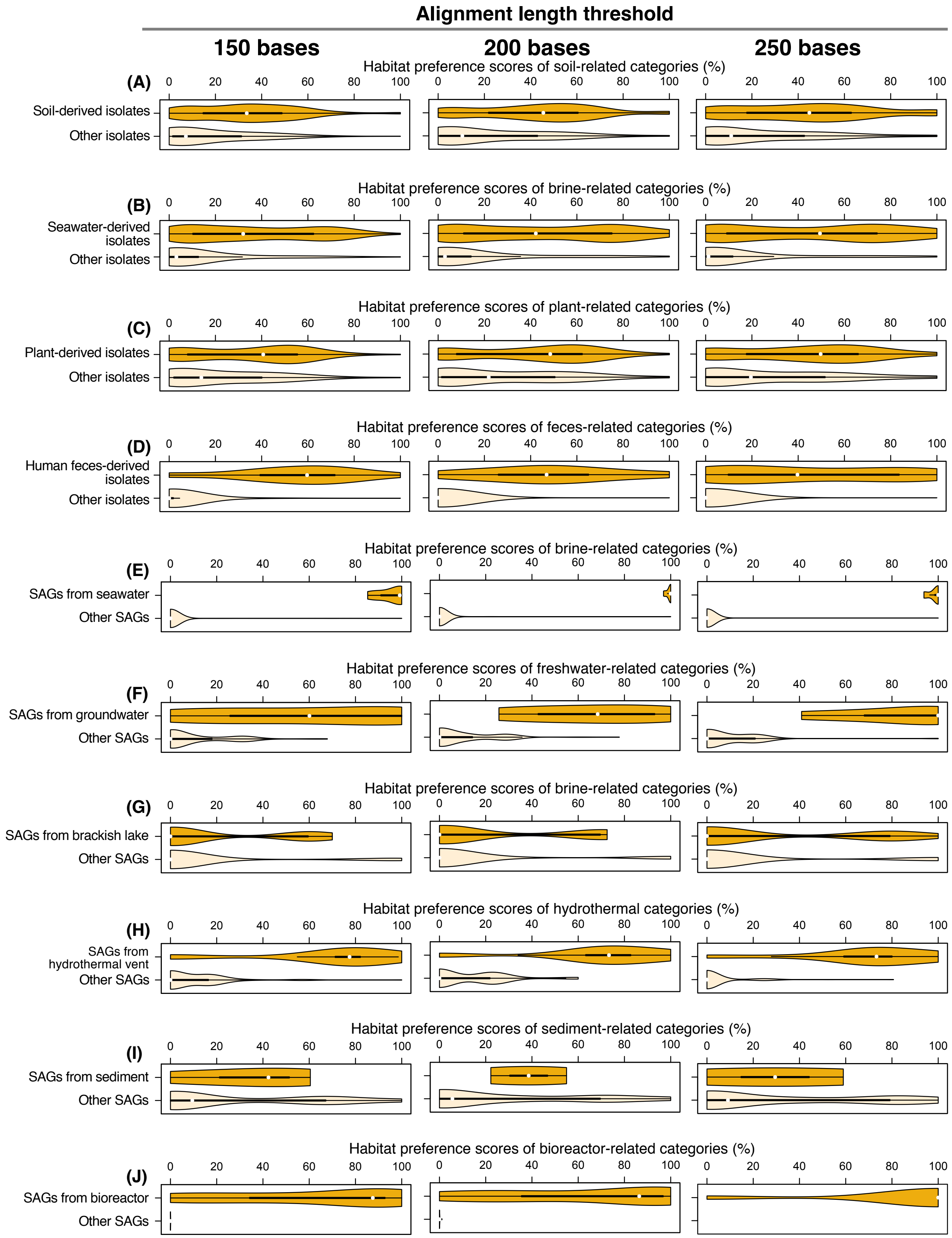

Figure S3. Violin plots indicating the effect of alignment length threshold on ProkAtlas-estimated prokaryotic habitat preference scores, Related to Figure 3 and the Transparent Methods

Panels in left, middle, and right columns show the results calculated with an alignment length threshold of 150 bases, 200 bases, and 250 bases, respectively. Details on each panel are explained in Figure 3.

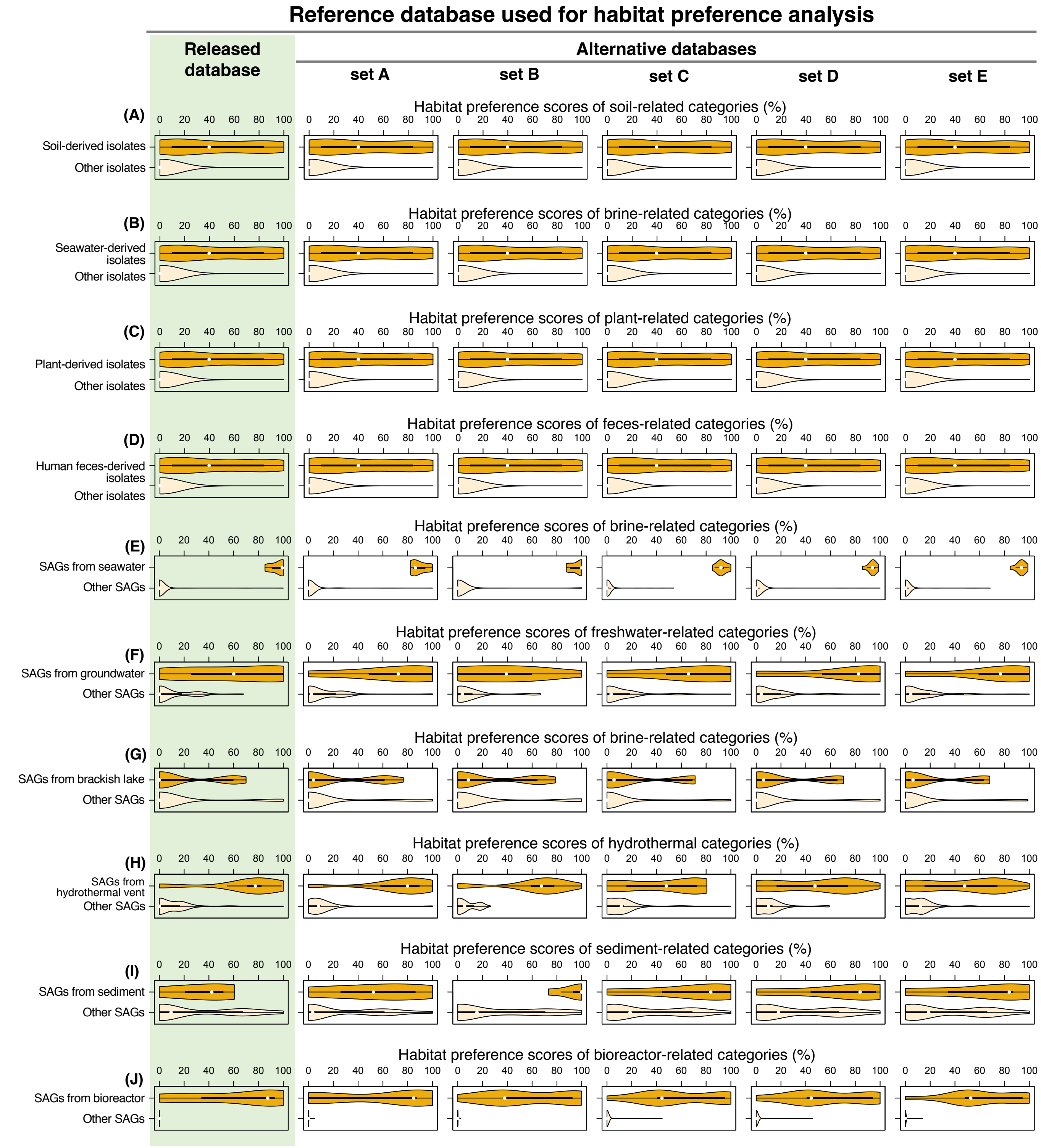

Figure S4. Violin plots indicating the effect of random sampling in constructing ProkAtlas on ProkAtlas-estimated prokaryotic habitat preference scores, Related to Figure 3 and the Transparent Methods

Panels in the leftmost column indicate the results obtained using the released version of ProkAtlas. Those in the next two columns indicate the results from alternative databases, each constructed by independent random sampling with at the same depth as ProkAtlas (up to 100 sequences per project). Panels in the last three columns indicate the results from yet other databases, each constructed by independent random sampling at deeper depth (up to 500 sequences per project). Details on each panel are explained in Figure 3.

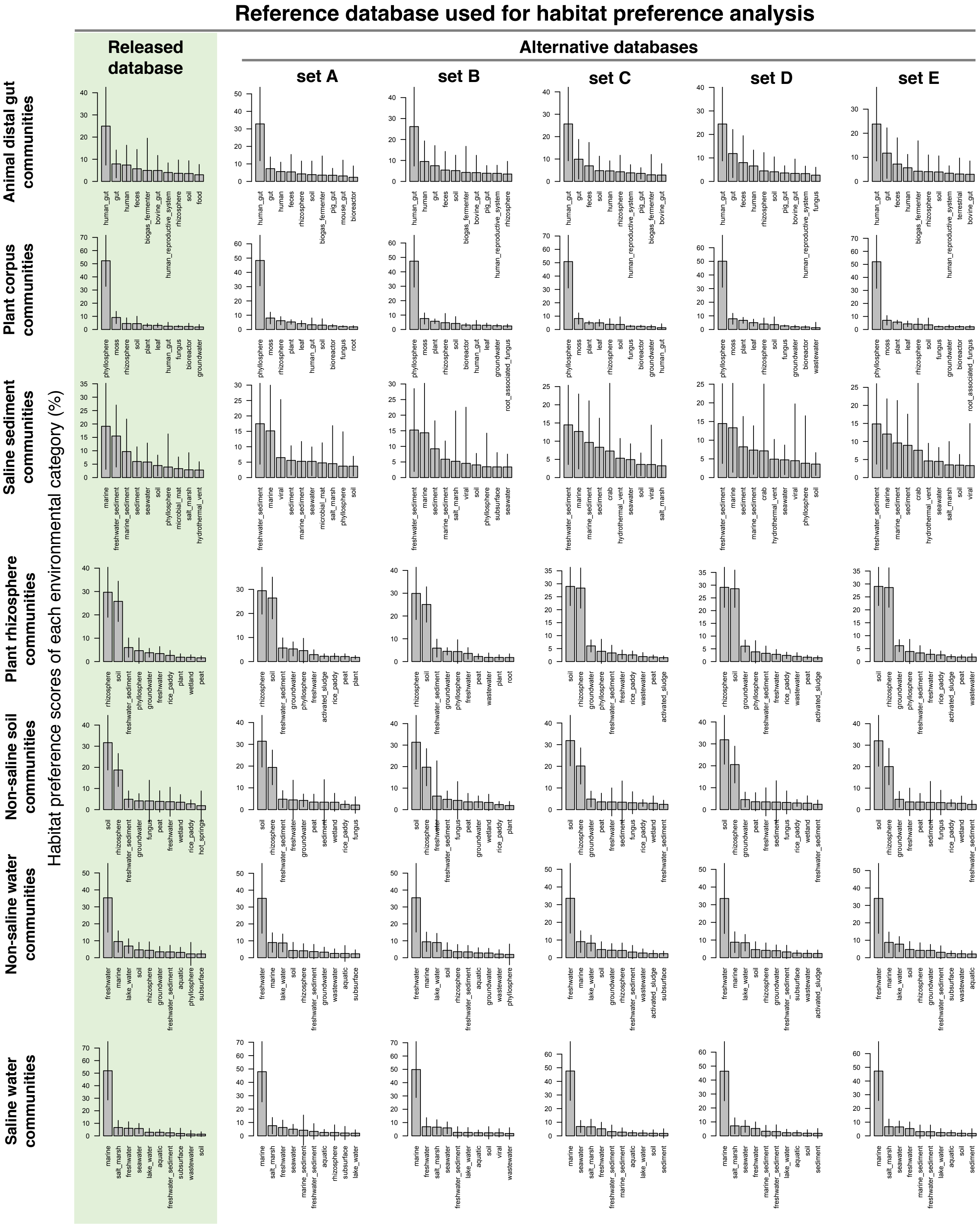

Figure S5. Habitat preference scores of EMP prokaryotic communities in each sampling site represented by EMP Ontology level 3 terms, using the released version of ProkAtlas and five alternative databases obtained by repeating the random sampling of sequences, Related to Figure 4 and the Transparent Methods

Details on each column and each panel are as explained in Figures S4 and 4, respectively.

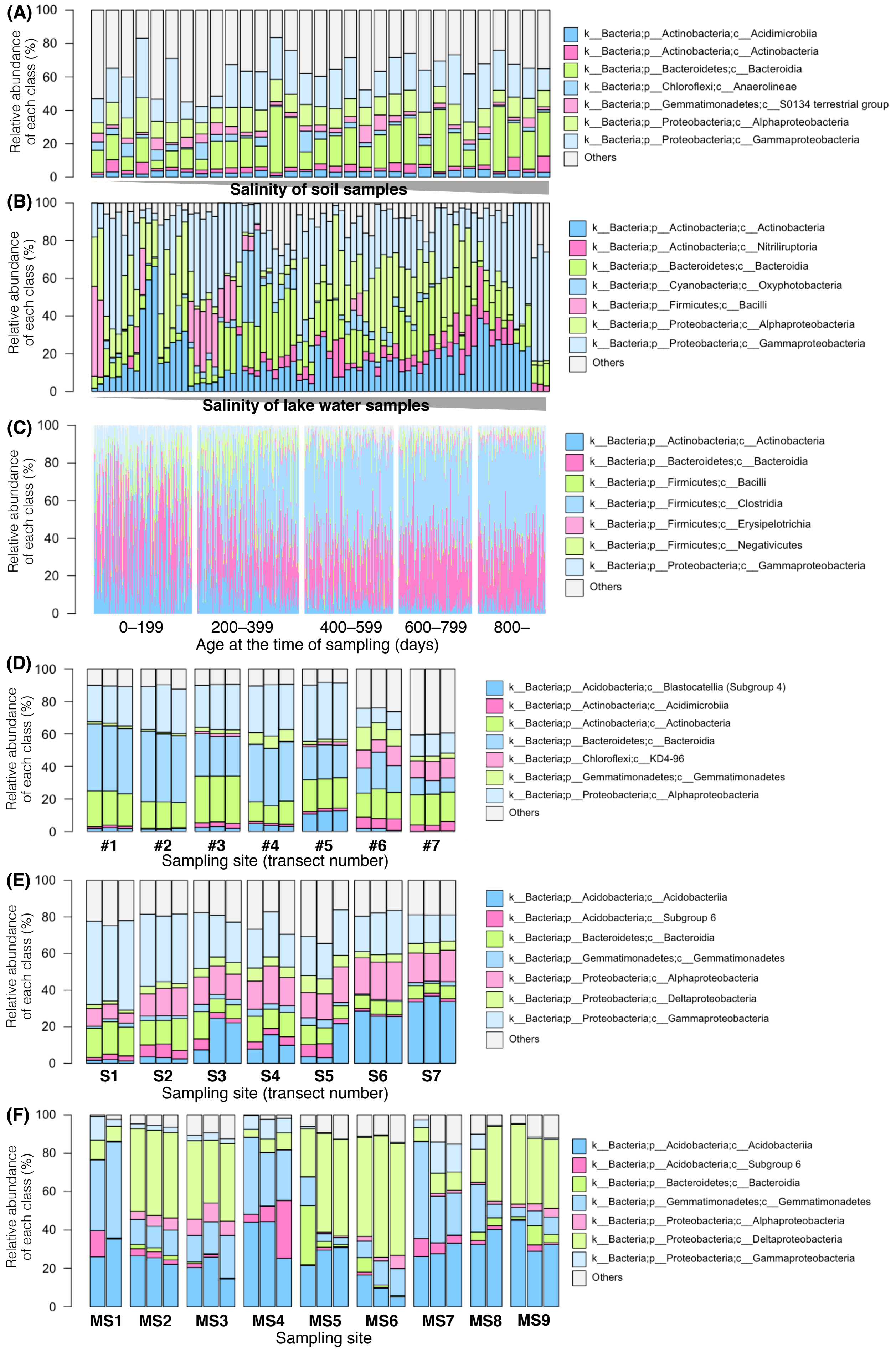
(A)
Relative abundance of each class (%)
k__Bacteria;p__Actinobacteria;c__Acidimicrobiia
k__Bacteria;p__Actinobacteria;c__Actinobacteria
k__Bacteria;p__Bacteroidetes;c__Bacteroidia
k__Bacteria;p__Chloroflexi;c__Anaerolineae
k__Bacteria;p__Gemmatimonadetes;c__S0134 terrestrial group
k__Bacteria;p__Proteobacteria;c__Alphaproteobacteria
k__Bacteria;p__Proteobacteria;c__Gammaproteobacteria
Others
Salinity of soil samples
(B)
Relative abundance of each class (%)
k__Bacteria;p__Actinobacteria;c__Actinobacteria
k__Bacteria;p__Actinobacteria;c__Nitriliruptoria
k__Bacteria;p__Bacteroidetes;c__Bacteroidia
k__Bacteria;p__Cyanobacteria;c__Oxyphotobacteria
k__Bacteria;p__Firmicutes;c__Bacilli
k__Bacteria;p__Proteobacteria;c__Alphaproteobacteria
k__Bacteria;p__Proteobacteria;c__Gammaproteobacteria
Others
Salinity of lake water samples
(C)
Relative abundance of each class (%)
k__Bacteria;p__Actinobacteria;c__Actinobacteria
k__Bacteria;p__Bacteroidetes;c__Bacteroidia
k__Bacteria;p__Firmicutes;c__Bacilli
k__Bacteria;p__Firmicutes;c__Clostridia
k__Bacteria;p__Firmicutes;c__Erysipelotrichia
k__Bacteria;p__Firmicutes;c__Negativicutes
k__Bacteria;p__Proteobacteria;c__Gammaproteobacteria
Others
0–199
200–399
400–599
600–799
800–
Age at the time of sampling (days)
(D)
Relative abundance of each class (%)
k__Bacteria;p__Acidobacteria;c__Blastocatellia (Subgroup 4)
k__Bacteria;p__Actinobacteria;c__Acidimicrobiia
k__Bacteria;p__Actinobacteria;c__Actinobacteria
k__Bacteria;p__Bacteroidetes;c__Bacteroidia
k__Bacteria;p__Chloroflexi;c__KD4-96
k__Bacteria;p__Gemmatimonadetes;c__Gemmatimonadetes
k__Bacteria;p__Proteobacteria;c__Alphaproteobacteria
Others
#1
#2
#3
#4
#5
#6
#7
Sampling site (transect number)
(E)
Relative abundance of each class (%)
k__Bacteria;p__Acidobacteria;c__Acidobacteriia
k__Bacteria;p__Acidobacteria;c__Subgroup 6
k__Bacteria;p__Bacteroidetes;c__Bacteroidia
k__Bacteria;p__Gemmatimonadetes;c__Gemmatimonadetes
k__Bacteria;p__Proteobacteria;c__Alphaproteobacteria
k__Bacteria;p__Proteobacteria;c__Deltaproteobacteria
k__Bacteria;p__Proteobacteria;c__Gammaproteobacteria
Others
S1
S2
S3
S4
S5
S6
S7
Sampling site (transect number)
(F)
Relative abundance of each class (%)
k__Bacteria;p__Acidobacteria;c__Acidobacteriia
k__Bacteria;p__Acidobacteria;c__Subgroup 6
k__Bacteria;p__Bacteroidetes;c__Bacteroidia
k__Bacteria;p__Gemmatimonadetes;c__Gemmatimonadetes
k__Bacteria;p__Proteobacteria;c__Alphaproteobacteria
k__Bacteria;p__Proteobacteria;c__Deltaproteobacteria
k__Bacteria;p__Proteobacteria;c__Gammaproteobacteria
Others
MS1
MS2
MS3
MS4
MS5
MS6
MS7
MS8
MS9
Sampling site

Figure S6. Class-level taxonomic structures of prokaryotic communities re-analyzed in this study, Related to Figures 5–9

(A) Class-level taxonomic structures of the 31 plots ordered by soil salinity concentration (higher on the right than on the left). Related to Figure 5. (B) Class-level taxonomic structures of the 76 samples ordered by salinity (higher on the right than on the left). Related to Figure 6. (C) Class-level taxonomic structures of the 654 samples ordered by sampling ages (older on the right). Related to Figure 7. (D) Class-level taxonomic structures in soil samples taken from Midtre Lovénbreen glacier moraine. Related to Figure 8. (E) Class-level taxonomic structures in soil samples taken from Hailuogou Glacier Chronosequence. Related to Figure 8. (F) Class-level taxonomic structures at nine sampling points (more downstream on the right than on the left). Related to Figure 9.

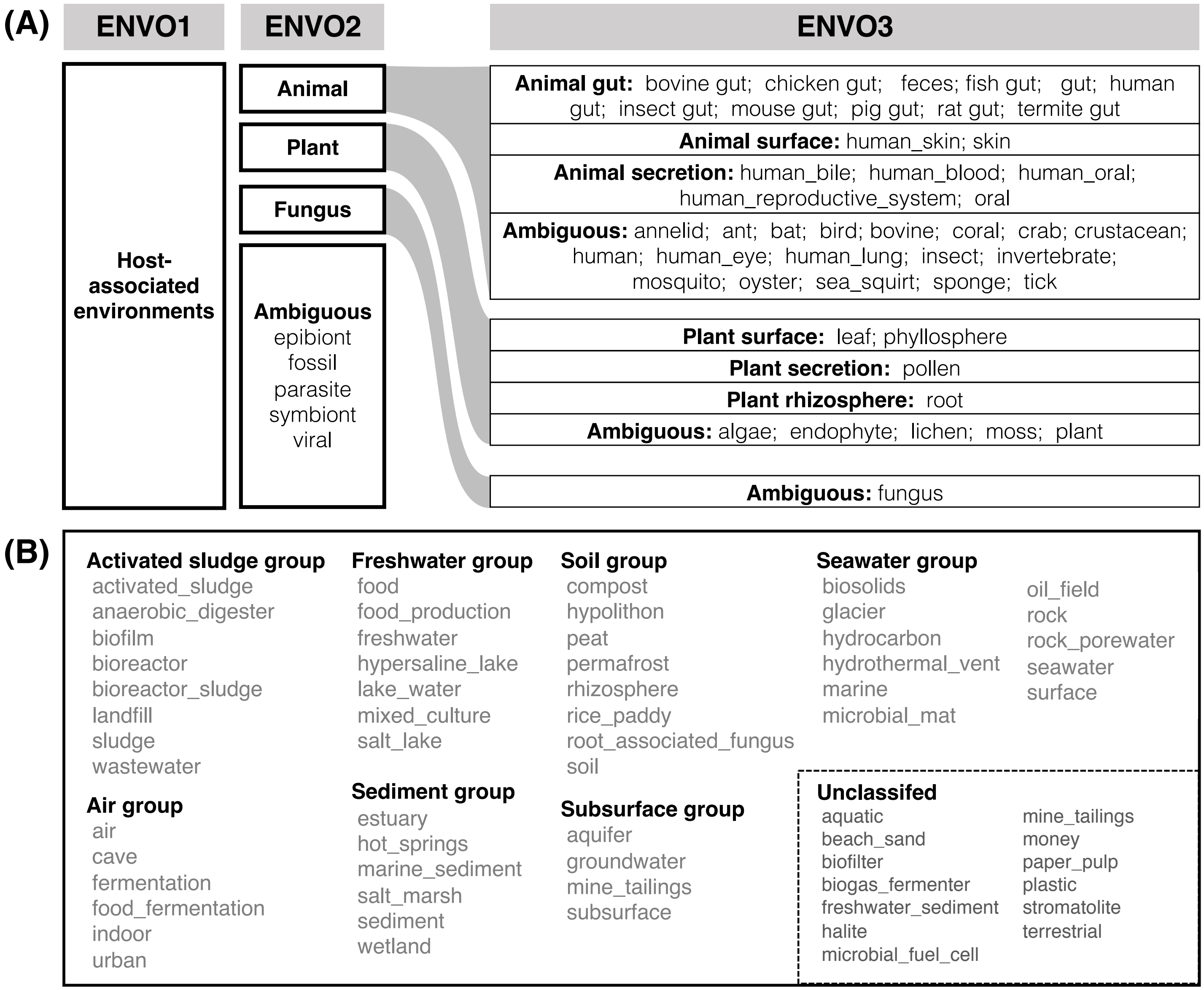

Figure S7. An example of classification of environmental categories, Related to Table 1

(A) Manual classification of host-associated environmental categories based on the Earth Microbiome Project Ontology (EMPO). (B) Classification of host-free environmental categories using random forest modeling of word use frequency in abstract texts attached to each DRA/ERA/SRA project. The modeling was performed using randomForest package implemented in R 3.6.2. The environmental categories were classified using a random forest classifier trained with abstracts from seven representative environments: *activated_sludge*, *air*, *freshwater*, *seawater*, *sediment*, *soil*, and *subsurface* (default parameters were used, except for the sampling size set to a maximum of 632 for each group). When none of the seven groups were assigned to >50% of the projects within an environmental category, that environmental category was assumed to be highly heterogenous (i.e. consisting of essentially different types of samples) and therefore left unclassified. For example, abstract texts of *beach_sand* samples were similar to both soil and seawater ones and therefore this category was regarded unclassifiable.

Transparent Methods

ProkAtlas database construction: Data source

We obtained all metagenomic sequence entries in DRA/ERA/SRA with 115 environmental categories (NCBI taxon IDs) under 410657, ecological metagenomes, and 410656, organismal metagenomes, on July 4, 2018. These entries contained metagenomic data from diverse environments and were based on different sequencing platforms and library construction strategies. We selected entries annotated as whole-genome sequencing (WGS) (i.e., shotgun sequencing data) to avoid PCR-biased data due to amplicon sequencing (Klindworth et al., 2012). We further selected entries generated by the most popular platform, i.e., Illumina sequencers, but excluded entries generated by HiSeq 3000, 4000, or X because of their potential inaccuracies (Sinha et al., 2017). The filtered entries included 5,368 projects, which contained 1–3,693 runs each (listed in Table S2). To avoid datasets that were too biased towards data from specific projects with high number of samples (Ramirez et al., 2018) and to keep the database size small, up to ten runs were randomly selected from each project. For each single-end or paired-end sequencing run, one or two gzipped fastq file(s), respectively, were downloaded from the ftp server of the European Nucleotide Archive (ENA). In the rare case in which a gzipped fastq file exceeded the data size of 200 MB, the first 200 MB was retrieved. The final dataset list is provided in Table S3.

ProkAtlas database construction: Data processing

Paired-end sequences with overlapping regions of 20 bp or longer were merged using USERACH v11.0.667 (Edgar, 2010), while single-end sequences were used as they were. Low quality regions (Q-score < 20) at the 3'-ends were pruned, and sequences with mean Q-scores of less than 30 were discarded using PRINSEQ 0.20.4 (Schmieder and Edwards, 2011). PARTIE (Torres et al., 2017) was used to remove amplicon sequence files mistakenly annotated as WGS. Sequences longer than 600 bases (the maximum read length of Illumina MiSeq and HiSeq) were removed, because they were likely artifacts. SortMeRNA 2.1 (Kopylova et al., 2012) trained with SILVA v132 SSU Nr99 (Quast et al., 2013) (hereafter referred to as SILVA) with the default parameter settings was used to extract 16S rRNA gene regions from query sequences. From the SILVA database used in this study, eukaryotic sequences (i.e. those annotated as "Eukaryota" at the kingdom/domain level) had been removed beforehand, retaining only prokaryotic 16S rRNA gene sequences. To filter out non-16S rRNA hits that mingled in the output sequences from SortMeRNA, they were further subjected to a BLASTn (BLAST+ 2.3.0) search against SILVA with an e-value threshold of 1E−10. For each query sequence, an alignment covering the longest part of query sequence was selected among the top 100 hits (in bitscore), and the aligned region of that query sequence was retrieved. When multiple hits tie in alignment length, one with the highest bitscore was chosen. If the longest-aligned query region was shorter than 150 bases excluding gaps, that sequence was removed. Because the number of rRNA gene sequences varied between projects (between 1 and 43,259 per project), we randomly sampled up to 100 sequences from each project. This prevented the datasets from being biased towards data from specific big projects, while keeping the database size small. In total, we compiled 361,474 rRNA gene sequences, each retaining environmental category information (Tables 1 and S1) that was accompanied by the original sequence dataset in DRA/ERA/SRA as a taxon ID. Note that each sequence in ProkAtlas is labeled by one environmental

category. To test if the randomness of the sampling step affects results and if the sampling of 100 sequences from each project is enough, we prepared five additional alternative datasets: two by sampling 100 sequences (sets A and B) and three by sampling 500 sequences (sets C, D, and E; each contained 1,412,963 rRNA gene sequences).

ProkAtlas pipeline

For habitat-based analysis of 16S rRNA gene sequence data, the ProkAtlas pipeline projects the associated environmental category data in ProkAtlas (originally presented as taxon IDs in DRA/ERA/SRA) to query sequences. A query can be either a single sequence from individual prokaryotic genome or a prokaryotic community dataset consisting of OTUs (or sub-OTUs, amplicon sequence variants) representative sequences and an OTU table (typically from amplicon and shotgun metagenomic sequencing). The ProkAtlas pipeline characterizes each query sequence or community with habitat preference scores, a vector denoting the composition of possible habitats inferred from compiled metagenomic sequences. A schematic illustration of the ProkAtlas pipeline is provided as Figure S2. The pipeline consists of two parts, namely BLASTn search against ProkAtlas database and calculation of habitat preference scores based on the hits retrieved by the BLASTn search.

The ProkAtlas pipeline uses a BLASTn search to query each input sequence against ProkAtlas, and all hits under an e-value threshold of 1E−5 are collected (the other parameters are set to default). Partial alignments are accepted because both query sequences and ProkAtlas entries can contain partial 16S rRNA genes (Figure S1A–D); however, hits harboring mismatches longer than 2 bp at either end of the alignment (Figure S1EF) are ignored because they may be erroneous hits. The hits are further filtered to satisfy sequence similarity and alignment length criteria (default: sequence similarity of 97% or more, alignment length of 150 bp or longer).

The habitat preference of a prokaryote or a prokaryotic community can be represented by a composition of environmental categories within the list of significant hits (Figure S2); however, simply counting a number of hits that are labeled with each environmental category may incorrectly emphasize hits to environments that are frequently studied, such as human gut. Therefore, the contribution of each environmental category is weighted by the log-transformed reciprocal of the proportion of sequences in that category within ProkAtlas (Yang and Iwasaki, 2014). This diminishes and increases the habitat preference scores of overrepresented and underrepresented categories, respectively.

Mathematically, a habitat preference score of a prokaryote or a prokaryotic community for each environmental category is defined by:

$$\mathbb{E}_i = \frac{\left(n_{soil}^i, n_{marine}^i, n_{gut}^i, \dots\right)}{\sum\left(n_{environment}^i\right)};$$

$$\mathbb{E} = \sum_i (C_i \times \mathbb{E}_i) \Big/ \sum_i C_i\,;$$

$$\mathbb{W} = \left(\log\left(\frac{R_{tot}}{R_{soil}}\right), \log\left(\frac{R_{tot}}{R_{marine}}\right), \log\left(\frac{R_{tot}}{R_{gut}}\right), \dots\right);$$

$$habitat\ preference\ score = \frac{\mathbb{E} \circ \mathbb{W}}{\sum(\mathbb{E} \circ \mathbb{W})}$$

where n_X^i is the number of significant hits to OTU *i* within a specific environmental category X, $\mathbb{E}_i$ is the environmental vector denoting the habitat preference of OTU *i*, C_i is the read count of OTU *i* (i.e. the

number of reads assigned to OTU i), $\mathbb{E}$ is the average of environmental vectors weighted by the read count of each OTU (i.e. C_i), R_{tot} and R_X are the number of ProkAtlas entries in total and within the environmental category X, respectively, and $\mathbb{W}$ is the vector of weighing factors of each environmental category. The arithmetic operator $\circ$ indicates the element-wise multiplication of two vectors with the same length (Hadamard product). When applied to a single prokaryotic sequence to illustrate the habitat preference of the corresponding microbe rather than community characteristics, the query is treated as a community composed of one OTU and one read (i.e., $\mathbb{E} = \mathbb{E}_i$).

The ProkAtlas database and pipeline are available at https://msk33.github.io/prokatlas.html.

Bird's-eye visualization of prokaryote cooccurence network

Because we constructed ProkAtlas using shotgun metagenomic sequences only, each of the sequences in ProkAtlas covers different regions of 16S rRNA genes. To compare these staggered sequences, they were mapped to SILVA using BLASTn search and subjected to closed-reference clustering. More specifically, up to 100 top hits (ranked by bitscores) were retrieved after the BLASTn search. Following the principle of parsimony, the greedy algorithm was employed to obtain the (approximately) smallest subset of SILVA entries containing at least one top hit for every query sequence (Chvatal, 1979). Then, for each environmental category, the number of sequences associated with each SILVA entry was counted. Of the 115 environmental categories, 27 categories harboring more than 2,000 sequences successfully mapped to SILVA were subjected to visualization. Bray-Curtis dissimilarities between the SILVA entry composition vectors associated with the environmental categories and betweenness centralities were calculated and their network was visualized using the *sna* package on R ver3.5.1 (R Core Team, 2017).

Application to 16S rRNA gene sequences of isolated and non-isolated prokaryotes

We downloaded 16S rRNA gene sequences of pure-isolated bacterial strains from manually curated IJSEM phenotypic database (https://doi.org/10.6084/m9.figshare.427239, as of October 2018) (Barberán et al., 2017). In addition, we downloaded 16S rRNA gene sequences produced from a large SAG sequencing project (Rinke et al., 2013). The ProkAtlas pipeline with the default parameter settings was used for habitat estimation, with an exception that we used three different alignment length thresholds, namely 150 (default value), 200, and 250 bases, to check the robustness of the pipeline. In addition, to test whether the random sampling process in constructing ProkAtlas affects the results, we performed the same analysis using the five alternative datasets as described above.

For each set of estimated habitat compositions, consistency with the source-environment information was tested. To test if estimated habitat compositions of soil-derived isolates are actually soil-related, the scores of environmental categories related to soil (namely "soil", "rhizosphere", "rice paddy", and "wetland") were compared between soil-derived and other isolates using the Mann-Whitney U-test.

Habitat-based analyses of prokaryotic community structure datasets

ProkAtlas was applied to datasets of EMP, saline-affected agricultural soil and lake water samples, human infant gut microbiome samples, glacier chronosequence soil samples, and potentially polluted

river-water samples. Notably, these were 16S rRNA gene amplicon-sequencing data and not included in ProkAtlas.

EMP data were downloaded from the EMP ftp server in February 2019 (Thompson et al., 2017). The data were based on the random picking of 2,000 samples and that of 5,000 sequences per sample (ftp://ftp.microbio.me/emp/release1/otu_tables/deblur/emp_deblur_150bp.subset_2k.rare_5000.biom). sOTUs clustered by Deblur (Amir et al., 2017) were used. Regarding the agricultural soil (Zhao et al., 2020), saline and non-saline lake water (Ji et al., 2019), infant gut microbiome (Yassour et al., 2016), glacier chronosequence soil (Jiang et al., 2018; Mapelli et al., 2018), and potentially polluted river-water (Kirs et al., 2017), raw fastq sequence data were downloaded from public databases (Table 2). Paired-end sequences with overlapping regions of 20 bp or longer were merged and quality-filtered using USEARCH v8.0.1623 (Edgar, 2010) (sequences with expected errors of 0.5 bp or less were kept), followed by removal of primer regions. sOTUs clustered by Deblur (Amir et al., 2017) with the default parameter settings were used.

The sOTUs were taxonomically annotated using RDP classifier (Wang et al., 2007) trained with SILVA with a confidence value threshold of 0.5. For one of the glacier chronosequence soil datasets (Mapelli et al., 2018), sOTUs annotated as members of phylum *Cyanobacteria* were eliminated because some samples were covered by cyanobacterial mat (Mapelli et al., 2018). Then, the sOTUs in each dataset were subjected to ProkAtlas pipeline, attributing each prokaryotic community to its estimated habitat composition. Regarding EMP dataset, which consists of short sequences (150 bases, the same as the default alignment length threshold), alignment length thresholds were set to 140 bases.

Supplemental References

Amir, A., Daniel, M., Navas-Molina, J., Kopylova, E., Morton, J., Xu, Z.Z., Eric, K., Thompson, L., Hyde, E., Gonzalez, A., et al. (2017). Deblur rapidly resolves single-nucleotide community sequence patterns. mSystems 2, e00191–16.

Chvatal, V. (1979). A Greedy Heuristic for the Set-Covering Problem. Math. Oper. Res. 4, 233.

Edgar, R.C. (2010). Search and clustering orders of magnitude faster than BLAST. Bioinformatics 26, 2460–2461.

Kopylova, E., Noé, L., and Touzet, H. (2012). SortMeRNA: Fast and accurate filtering of ribosomal RNAs in metatranscriptomic data. Bioinformatics 28, 3211–3217.

Quast, C., Pruesse, E., Yilmaz, P., Gerken, J., Schweer, T., Yarza, P., Peplies, J., and Glöckner, F.O. (2013). The SILVA ribosomal RNA gene database project: Improved data processing and web-based tools. Nucleic Acids Res. 41, 590–596.

R Core Team, 2017. R: A language and environment for statistical computing. R Foundation for Statistical Computing, Vienna, Austria. URL https://www.R-project.org/.

Schmieder, R. and Edwards, R.A. (2011). Quality control and preprocessing of metagenomic datasets. Bioinformatics 27, 863–864.

Sinha, R., Stanley, G., Gulati, G.S., Ezran, C., Travaglini, K.J., Wei, E., Chan, C.K.F., Nabhan, A.N., Su, T., Morganti, R.M., et al. (2017). Index switching causes “spreading-of-signal” among multiplexed samples in Illumina HiSeq 4000 DNA sequencing. bioRxiv 125724. https://doi.org/10.1101/125724

Torres, P.J., Edwards, R.A., and McNair, K.A. (2017). PARTIE: A partition engine to separate metagenomic and amplicon projects in the Sequence Read Archive. Bioinformatics 33, 2389–2391.

Wang, Q., Garrity, G.M., Tiedje, J.M., and Cole, J.R. (2007). Naive Bayesian classifier for rapid assignment of rRNA sequences into the new bacterial taxonomy. Appl. Environ. Microbiol. 73, 5261–5267.
